# Retrospective observation on contribution and limitations of screening for breast cancer with mammography in Korea: detection rate of breast cancer and incidence rate of interval cancer of the breast

**DOI:** 10.1186/s12905-016-0351-1

**Published:** 2016-11-18

**Authors:** Kunsei Lee, Hyeongsu Kim, Jung Hyun Lee, Hyoseon Jeong, Soon Ae Shin, Taehwa Han, Young Lan Seo, Youngbum Yoo, Sang Eun Nam, Jong Heon Park, Yoo Mi Park

**Affiliations:** 1 Departments of Preventive Medicine, School of Medicine, Konkuk University, 1 Hwayang-dong, Gwangjin-gu, Seoul, 143-701 South Korea; 2 Bigdata Steering Department, National Health Insurance Service, Wonju, South Korea; 3 Yonsei University Health System, College of Medicine, Yonsei University, Seoul, South Korea; 4 Department of Radiology, Kangdong Seong-Sim Hospital, College of Medicine, Hallym University, Seoul, South Korea; 5 Departments of Surgery, School of Medicine, Konkuk University, Seoul, South Korea; 6 Medical and Health Policy Division, Seoul Metropolitan Government, Seoul, South Korea

**Keywords:** Screening, Breast cancer, Mammography

## Abstract

**Background:**

The purpose of this study was to determine the benefits and limitations of screening for breast cancer using mammography.

**Methods:**

Descriptive design with follow-up was used in the study. Data from breast cancer screening and health insurance claim data were used. The study population consisted of all participants in breast cancer screening from 2009 to 2014. Crude detection rate, positive predictive value and sensitivity and specificity of breast cancer screening and, incidence rate of interval cancer of the breast were calculated.

**Results:**

The crude detection rate of breast cancer screening per 100,000 participants increased from 126.3 in 2009 to 182.1 in 2014. The positive predictive value of breast cancer screening per 100,000 positives increased from 741.2 in 2009 to 1,367.9 in 2014. The incidence rate of interval cancer of the breast per 100,000 negatives increased from 51.7 in 2009 to 76.3 in 2014. The sensitivities of screening for breast cancer were 74.6% in 2009 and 75.1% in 2014 and the specificities were 83.1% in 2009 and 85.7% in 2014.

**Conclusions:**

To increase the detection rate of breast cancer by breast cancer screening using mammography, the participation rate should be higher and an environment where accurate mammography and reading can be performed and reinforcement of quality control are required. To reduce the incidence rate of interval cancer of the breast, it will be necessary to educate women after their 20s to perform self-examination of the breast once a month regardless of participation in screening for breast cancer.

## Background

Malignant neoplasm of the breast has a major influence on the death rate among women; it is the cancer with the second highest incidence rate in Korean women after malignant neoplasm of the thyroid. The crude incidence rate of malignant neoplasm of the breast in women increased from 24.3 persons per 100,000 population in 1999 to 65.7 persons per 100,000 population in 2012 [1].

For early detection of breast cancer, the National Health Insurance Service (NHIS) in Korea began screening for breast cancer using mammography and physical examination of the breast concurrently once every 2 years without copayment in 1999, targeting women with medical aid who were over 40 years of age [2]. Free screening for breast cancer was expanded to include those with health insurance who were in the lowest 20% of income in 2002, the lowest 30% in 2003, and lower 50% in 2005; currently, even those who do not qualify for free breast cancer screening may receive screening by paying part of the cost (10% since 2010). The expansion of eligibility for screening and reduction of screening cost resulted in increases in the breast cancer screening participation rate from 23.6% (4,437,492 individuals) in 2005 to 59.3% (5,849,134 individuals) in 2014 [3].

Recently, the committee that developed the Korean guideline for breast cancer screening concluded that the benefits of screening for breast cancer with mammography outweighed the potential harm based on the results of meta-analysis using the domestic and foreign literature on death rate, total death rate, and stage shift of breast cancer and they issued the Korean guidelines for breast cancer screening, encouraging women ages 40–69 to undergo breast cancer screening by mammography and physical examination every 2 years [4]. However, mammography has a number of drawbacks, including radiation exposure [5], overdiagnosis [6], anxiety due to false positive results [7], unnecessary biopsies and surgery [8], incidence of interval cancers [9], and psychological stress [10]. In addition, some researchers insist that people should make their own informed decision regarding breast cancer screening based on being provided sufficient information about the benefits and the potential harm, as breast cancer screening does not have a marked effect on the incidence of breast cancer and death [11, 12]. Nevertheless, mammography is the best radiological diagnostic screening tool from the cost–effectiveness viewpoint, and many countries and institutes encourage breast cancer screening using this method [13].

Although research on breast cancer screening has focused mainly on the intention and determinants of breast cancer screening [14, 15], the distribution of dense breast tissue and related factors in mammography [16, 17], and comparison of mammography and ultrasound examination [18, 19], there has been inadequate discussion regarding the benefits and limitations of breast cancer screening with mammography. This study was performed to determine the benefits and limitations of screening for breast cancer using mammography based on the detection rate, the positive predictive value of screening for breast cancer, and the incidence rate of interval breast cancer.

## Methods

### Study design and sample

Descriptive design with follow-up was used in the study. This study used data from breast cancer screening with mammography from January 2009 to December 2014, combined with health insurance claim data on medical expenses for breast cancer treatment from January 2002 to August 2015 extracted from the electronic data of NHIS.

The study population consisted of all participants in breast cancer screening conducted by NHIS over the 6 years from 2009 to 2014. Participants who had been treated for breast cancer (malignant neoplasm or carcinoma in situ) prior to screening were excluded. The final numbers of subjects included in the analyses were 2,977,041 in 2009, 2,907,964 in 2010, 3,359,526 in 2011, 3,334,657 in 2012, 3,291,981 in 2013, and 3,564,681 in 2014. Screening for breast cancer with mammography conducted by NHIS in Korea is currently carried out for all women over 40 years of age regardless of previous breast cancer diagnosis.

### Measurement

1. Selection and processing of variables
  Subjects’ ID, age, screening date, and screening results were selected from breast cancer screening data, and subjects’ ID, age, sex, disease code (including 1st to 5th diagnoses), treatment methods (anticancer drugs or operation), special case calculation, and the date of visits to medical institutions were selected from the health insurance claim data.
  The subjects were classified according to age at the time of screening as 40–49, 50–59, 60–69, and 70 years or older, and the screening results were classified as normal, benign lesion, suspected breast cancer, and deferred. Using the disease codes in the health insurance claim data, breast cancers were classified as malignant neoplasms, (C50 in the Korean Standard Classification of Diseases) or carcinoma in situ (D05).
  The detection of breast cancer was judged according to the use of anticancer drugs, surgery, such as mastectomy or radical mastectomy related to breast cancer, and special case calculations, as described below. That is, subjects were defined as having breast cancer if they were diagnosed with malignant neoplasm or carcinoma in situ, received anticancer medication related to breast cancer or breast cancer surgery and were the subject of special case calculation. The date of breast cancer detection was defined as the first visit day (hospitalization or outpatient) to a medical institution for anticancer drugs or surgery. For example, a breast cancer detection date of May 1, 2009, in the health insurance claim data indicated that the patient had received no medical treatment for breast cancer from January 1, 2002, to April 30, 2009, at an outpatient department or with hospitalization.
  Special case calculation is a system whereby the economic burden on patients diagnosed with cancer, cerebrovascular and heart diseases, severe burns, or intractable diseases is reduced by decreasing the copayment for treatment for the disease registered by NHIS for 5 years. This system was introduced in July 2001; the copayment was reduced from 20 to 10% in September 2005, and to 5% in December 2009. Malignant neoplasm and carcinoma in situ of the breast are included in the criteria for special case calculation.
2. Merging of breast cancer screening data and health insurance claim data
  Breast cancer screening data and evaluation of insurance claim data on breast cancer were merged according to the subjects’ ID.
3. Index for contributions and limitations of breast cancer screening

The index for the contributions and limitations of breast cancer screening in breast cancer detection and the basic items for calculation are as follows:

**Table:** 

		Breast cancer based on health insurance claim data		Total
		Detected	Not detected	
Results of screening for breast cancer	Positive	a	b	a + b
	Negative	c	d	c + d
Total		a + c	b + d	N

From the results of breast cancer screening, suspected and deferred breast cancer diagnoses were defined as positive screening outcomes, and test results showing normal findings and benign lesions as negative. Among the participants in breast cancer screening, if treatment for breast cancer occurred within 6 months from breast cancer screening, it was defined as detection by breast cancer screening; the absence of treatment for breast cancer within that period was defined as non-detection.

1. Positive rate of screening for breast cancer
  The positive rate of screening for breast cancer was defined as the number of positive tests (a + b) per 100 breast cancer screening participants (N).
  $$ \mathrm{Positive}\ \mathrm{r}\mathrm{ate}\ \mathrm{o}\mathrm{f}\ \mathrm{screening}\ \mathrm{f}\mathrm{o}\mathrm{r}\ \mathrm{breast}\ \mathrm{cancer}=\frac{\mathrm{Tested}\ \mathrm{positive}\ \left(\mathrm{a}+\mathrm{b}\right)}{\mathrm{Breast}\ \mathrm{cancer}\ \mathrm{screening}\ \mathrm{participants}\left(\mathrm{N}\right)}\times 100 $$
2. Crude detection rate for breast cancer screening
  The crude detection rate for breast cancer screening was defined as the number of subjects with positive test results in whom breast cancer was detected (a) per 100,000 breast cancer screening participants (N).
  $$ \mathrm{Crude}\ \mathrm{detection}\ \mathrm{r}\mathrm{ate}\ \mathrm{f}\mathrm{o}\mathrm{r}\ \mathrm{breast}\ \mathrm{cancer}\ \mathrm{screening}=\frac{\mathrm{Tested}\ \mathrm{positive}\ \mathrm{and}\ \mathrm{detected}\ \mathrm{breast}\ \mathrm{cancer}\left(\mathrm{a}\right)}{\mathrm{Breast}\ \mathrm{cancer}\ \mathrm{screening}\ \mathrm{participants}\left(\mathrm{N}\right)}\times 100,000 $$
3. Positive predictive value (PPV) of breast cancer screening
  The PPV of screening for breast cancer was defined as the number of detected breast cancer patients (a) per 100,000 who received positive results on breast cancer screening (a + b).
  $$ \mathrm{Positive}\ \mathrm{predictive}\ \mathrm{value}\ \mathrm{o}\mathrm{f}\;\mathrm{screening}\ \mathrm{f}\mathrm{o}\mathrm{r}\ \mathrm{breast}\ \mathrm{cancer}=\frac{\mathrm{Detected}\ \mathrm{breast}\ \mathrm{cancer}\ \left(\mathrm{a}\right)}{\mathrm{Positive}\ \mathrm{breast}\ \mathrm{cancer}\ \mathrm{screening}\ \left(\mathrm{a}+\mathrm{b}\right)}\times 100,000 $$
4. Incidence rate of interval cancer of the breast
  Cancers that occur within 12 months after negative results on cancer screening are termed interval cancers [20]. The incidence rate of interval cancer of the breast was defined as the number cases of breast cancer detected (c) among 100,000 with negative results on the breast cancer screening (c + d).
  $$ \mathrm{Incidence}\ \mathrm{rate}\ \mathrm{of}\ \mathrm{interval}\ \mathrm{cancer}\ \mathrm{of}\ \mathrm{the}\ \mathrm{breast}=\frac{\mathrm{Detected}\ \mathrm{breast}\ \mathrm{cancer}\left(\mathrm{c}\right)}{\mathrm{Negative}\ \mathrm{breast}\ \mathrm{cancer}\ \mathrm{screening}\ \mathrm{result}\left(\mathrm{c}+\mathrm{d}\right)}\times 100,000 $$
5. Sensitivity and specificity of screening for breast cancer

The sensitivity of screening for breast cancer was defined as number of positive breast cancer screening results (a) among 100 detected cases of breast cancer (a + c), and the specificity of screening for breast cancer as the number of negative results on breast cancer screening (d) among 100 cases where breast cancer was not detected (b + d).

$$ \mathrm{Sensitivity}\ \mathrm{o}\mathrm{f}\ \mathrm{screening}\ \mathrm{f}\mathrm{o}\mathrm{r}\ \mathrm{breast}\ \mathrm{cancer}=\frac{\mathrm{Positive}\ \mathrm{breast}\ \mathrm{cancer}\ \mathrm{screening}\ \mathrm{r}\mathrm{esult}\ \left(\mathrm{a}\right)}{\mathrm{Detected}\ \mathrm{breast}\ \mathrm{cancer}\ \left(\mathrm{a}+\mathrm{c}\right)}\times 100 $$

$$ \mathrm{Specificity}\ \mathrm{o}\mathrm{f}\ \mathrm{screening}\ \mathrm{f}\mathrm{o}\mathrm{r}\ \mathrm{breast}\ \mathrm{cancer}=\frac{\mathrm{Negative}\ \mathrm{breast}\ \mathrm{cancer}\ \mathrm{screening}\ \mathrm{r}\mathrm{esult}\ \left(\mathrm{d}\right)}{\mathrm{Breast}\ \mathrm{cancer}\ \mathrm{not}\ \mathrm{detected}\ \left(\mathrm{b}+\mathrm{d}\right)}\times 100 $$

### Data analysis

Data analyses were performed using SAS statistical software (ver. 9.1, SAS Institute, Cary, NC). Using the data of breast cancer screening and health insurance claim data, the study subjects were selected, and the positive rate, crude detection rate, PPV, sensitivity, and specificity of breast cancer screening and incidence rate of interval cancer of the breast were calculated.

## Results

1. Positive rate of screening for breast cancer
  The positive rate of screening for breast cancer decreased from 17.0% in 2009 to 14.8% in 2014 (Table 1). While the positive rates for women in their 40s and 50s during the same period decreased from 24.7% and 17.8 to 20.1% and 15.8%, respectively, those for women in their 60s and 70s increased from 10.6% and 5.9 to 11.3% and 7.8%, respectively. During all 6 years, based on women in their 40s, the positive rate decreased as age increased.

**Table 1:** Results of screening for breast cancer with mammography from 2009 to 2014

			2009		2010		2011		2012		2013		2014	
			No	%	No	%	No	%	No	%	No	%	No	%
Total participants			2,977,041	100.0	2,907,964	100.0	3,359,526	100.0	3,334,657	100.0	3,291,981	100.0	3,564,681	100.0
Results of screening	Negative	Subtotal	2,469,917	83.0	2,473,581	85.1	2,874,166	85.6	2,843,801	85.3	2,817,325	85.6	3,035,615	85.2
		Normal	2,163,673	87.6	2,121,346	85.8	2,465,337	85.8	2,426,471	85.3	2,378,842	84.4	2,560,714	84.4
		Benign lesion	306,244	12.4	352,235	14.2	408,829	14.2	417,330	14.7	438,483	15.6	474,901	15.6
	Positive	Subtotal	507,124	17.0	434,383	14.9	485,360	14.4	490,856	14.7	474,656	14.4	529,066	14.8
		Suspected breast cancer	6,249	1.2	5,384	1.2	5,588	1.2	5,253	1.1	4,762	1.0	4,649	0.9
		Deferred	500,875	98.8	428,999	98.8	479,772	98.8	485,603	98.9	469,894	99.0	524,417	99.1
Age	40–49	Subtotal	1,014,401	34.1	987,795	34.0	1,069,780	31.8	1,036,136	31.1	1,023,722	31.1	1,084,874	30.4
		Negative	763,858	75.3	775,653	78.5	853,005	79.7	823,571	79.5	825,570	80.6	866,479	79.9
		Positive	250,543	24.7	212,142	21.5	216,775	20.3	212,565	20.5	198,152	19.4	218,395	20.1
	50–59	Subtotal	941,260	31.6	922,458	31.7	1,133,029	33.7	1,098,477	32.9	1,082,582	32.9	1,146,449	32.2
		Negative	775,482	82.4	783,436	84.9	966,230	85.3	930,045	84.7	919,229	84.9	967,212	84.4
		Positive	165,778	17.6	139,022	15.1	166,899	14.7	168,432	15.3	163,353	15.1	179,237	15.6
	60–69	Subtotal	643,458	21.6	615,546	21.2	698,331	20.8	716,442	21.5	697,346	21.2	794,659	22.3
		Negative	575,039	89.4	555,855	90.3	627,843	89.9	640,571	89.4	621,607	89.1	705,162	88.7
		Positive	68,419	10.6	59,691	9.7	70,488	10.1	75,871	10.6	75,739	10.9	89,497	11.3
	Over 70	Subtotal	377,922	12.7	382,165	13.1	458,386	13.6	483,602	14.5	488,331	14.8	538,699	15.1
		Negative	355,538	94.1	358,637	93.8	427,088	93.2	449,614	93.0	450,919	92.3	496,762	92.2
		Positive	22,384	5.9	23,528	6.2	31,298	6.8	33,988	7.0	37,412	7.7	41,937	7.8
2. Crude detection rate of breast cancer screening
  The crude detection rate of breast cancer screening per 100,000 participants increased from 126.3 in 2009 to 182.1 in 2014 (Table 2). During the same period, the crude detection rate of carcinoma in situ increased from 28.0 to 42.3, and the crude detection rate of malignant neoplasm from 98.3 to 139.8. The crude detection rates of carcinoma in situ and of malignant neoplasm increased in all age groups during the same period. During all 6 years, the crude detection rates of carcinoma in situ and of malignant neoplasm were highest in women in their 40s and in their 50s, respectively.

**Table 2:** Detection rate and positive predictive rate of screening for breast cancer with mammography from 2009 to 2014

			2009				2010				2011				2012				2013				2014			
			No. of detection	%	cDR	PPV	No. of detection	%	cDR	PPV	No. of detection	%	cDR	PPV	No. of detection	%	cDR	PPV	No. of detection	%	cDR	PPV	No. of detection	%	cDR	PPV
Detected breast cancer		Total	3,759	100.0	126.3	741.2	3,953	100.0	135.9	910.0	4,696	100.0	139.8	967.5	5,324	100.0	159.7	1,084.6	5,676	100.0	172.4	1,195.8	6,493	100.0	182.1	1,367.9
		CIS	833	22.2	28.0	164.3	872	22.1	30.0	200.7	1,049	22.3	31.2	216.1	1,248	23.4	37.4	254.2	1,272	22.4	38.6	268.0	1,509	23.2	42.3	317.9
		Malignant N.	2,926	77.8	98.3	577.0	3,081	77.9	106.0	709.3	3,647	77.7	108.6	751.4	4,076	76.6	122.2	830.4	4,404	77.6	133.8	927.8	4,984	76.8	139.8	1,050.0
Age	40–49	Subotal	1,412	37.6	139.2	563.6	1,416	35.8	143.3	667.5	1,580	33.6	147.7	728.9	1,805	33.9	174.2	849.2	1,836	32.3	179.3	926.6	2,125	32.7	195.9	973.0
		CIS	353	25.0	34.8	140.9	355	25.1	35.9	167.3	393	24.9	36.7	181.3	479	26.5	46.2	225.3	448	24.4	43.8	226.1	570	26.8	52.5	261.0
		Malignant N.	1,059	75.0	104.4	422.7	1,061	74.9	107.4	500.1	1,187	75.1	111.0	547.6	1,326	73.5	128.0	623.8	1,388	75.6	135.6	700.5	1,555	73.2	143.3	712.0
	50–59	Subotal	1,344	35.8	142.8	810.7	1,476	37.3	160.0	1,061.7	1,701	36.2	150.1	1,019.2	1,909	35.9	173.8	1,133.4	2,124	37.4	196.2	1,300.3	2,370	36.5	206.7	1,322.3
		CIS	294	21.9	31.2	177.3	330	22.4	35.8	237.4	381	22.4	33.6	228.3	465	24.4	42.3	276.1	485	22.8	44.8	296.9	533	22.5	46.5	297.4
		Malignant N.	1,050	78.1	111.6	633.4	1,146	77.6	124.2	824.3	1,320	77.6	116.5	790.9	1,444.	75.6	131.5	857.3	1,639	77.2	151.4	1,003.3	1,837	77.5	160.2	1,024.9
	60–69	Subotal	728	19.4	113.1	1,064.0	744	18.8	120.9	1,246.4	1,003	21.4	143.6	1,422.9	1,098	20.6	153.3	1,447.2	1,091	19.2	156.5	1,440.5	1,371	21.1	172.5	1,531.9
		CIS	142	19.5	22.1	207.5	142	19.1	23.1	237.9	212	21.1	30.4	300.8	222	20.2	31.0	292.6	223	20.4	32.0	294.4	286	20.9	36.0	319.6
		Malignant N.	586	80.5	91.1	856.5	602	80.9	97.8	1,008.5	791	78.9	113.3	1,122.2	876	79.8	122.3	1,154.6	868	79.6	124.5	1,146.0	1,085	79.1	136.5	1,212.3
	70	Subotal	275	7.3	72.8	1,228.6	317	8.0	82.9	1,347.3	412	8.8	89.9	1,316.4	512	9.6	105.9	1,506.4	625	11.0	128.0	1,670.6	627	9.7	116.4	1,495.1
		CIS	44	16.0	11.6	196.6	45	14.2	11.8	191.3	63	15.3	13.7	201.3	82	16.0	17.0	241.3	116	18.6	23.8	310.1	120	19.1	22.3	286.1
		Malignant N.	231	84.0	61.1	1,032.0	272	85.8	71.2	1,156.1	349	84.7	76.1	1,115.1	430	84.0	88.9	1,265.2	509	81.4	104.2	1,360.5	507	80.9	94.1	1,209.0
Results of screening	Suspected breast cancer	Subotal	1,252	33.3	–	20,035.2	1,290	32.6	–	23,959.9	1,439	30.6	–	25,751.6	1,624	30.5	-	30,915.7	1,655	29.2	-	34,754.3	1,803	27.8	-	37,862.2
		CIS	225	18.0	–	3,600.6	230	17.8	–	4,271.9	271	18.8	–	4,849.7	307	18.9	-	5,844.3	289	17.5	-	6,068.9	310	17.2	-	6,509.9
		Malignant N.	1,027	82.0	–	16,434.6	1,060	82.2	–	19,688.0	1,168	81.2	–	20,901.9	1,317	81.1	–	25,071.4	1,366	82.5	–	28,685.4	1,493	82.8	–	31,352.4
	Deferred	Subotal	2,507	66.7	–	500.5	2,663	67.4	–	620.7	3,257	69.4	–	678.9	3,700	69.5	–	761.9	4,021	70.8	–	855.7	4,690	72.2	–	998.1
		CIS	608	24.3	–	121.4	642	24.1	–	149.7	778	23.9	–	162.2	941	25.4	–	193.8	983	24.4	–	209.2	1,199	25.6	–	255.2
		Malignant N.	1,899	75.7	–	379.1	2,021	75.9	–	471.1	2,479	76.1	–	516.7	2,759	74.6	–	568.2	3,038	75.6	–	646.5	3,491	74.4	–	742.9

*cDR* crude detection rate, those who were tested positive and detected breast cancer patients among 100,000 breast cancer screening participants, *PPV* Positive predictive value, the detected breast cancer patient among 100,000 positives of breast cancer screening, *CIS* Carcinoma in situ of breast, *Malignant N* Malignant neoplasm of breast
3. PPV of screening for breast cancer
  The PPV of breast cancer screening per 100,000 positives increased from 741.2 in 2009 to 1,367.9 in 2014 (Table 2). During the same period, the PPV of carcinoma in situ increased from 164.3 to 317.9, and the PPV of malignant neoplasm increased from 577.0 to 1,050.0. The PPV of carcinoma in situ and of malignant neoplasm increased in all age groups during the same period. During all 6 years, the PPV of carcinoma in situ and of malignant neoplasm were highest in women in their 50s and in their 60s, respectively.
  In breast cancer screening, the PPV of suspected breast cancer increased from 20,035.2 in 2009 to 37,862.2 in 2014, and the PPV of cases classified as deferred increased from 500.5 to 998.1 during the same period.
4. Incidence rate of interval cancer in breast cancer screening
  The incidence rate of interval cancer of the breast per 100,000 negatives increased from 51.7 in 2009 to 76.3 in 2014 (Table 3). During the same period, the incidence rate of interval carcinoma in situ increased from 9.9 to 17.8, and that of interval malignant neoplasm increased from 41.8 to 58.5. The incidence rates of interval carcinoma in situ and of interval malignant neoplasm increased in all age groups during the same period. During all 6 years, the incidence rates of both interval carcinoma in situ and of interval malignant neoplasm were highest in women in their 40s.

**Table 3:** Incidence rate of breast interval cancer from 2009 to 2014

			2009			2010			2011			2012			2013			2014		
			No. of incidence	%	IR	No. of incidence	%	IR	No. of incidence	%	IR	No. of incidence	%	IR	No. of incidence	%	IR	No. of incidence	%	IR
Breast interval cancer		Total	1,278	100.0	51.7	1,385	100.0	56.0	1,801	100.0	62.7	1,912	100.0	67.2	1,986	100.0	70.5	2,150	100.0	76.3
		CIS	245	19.2	9.9	259	18.7	10.5	392	21.8	13.6	436	22.8	15.3	446	22.5	15.8	501	23.3	17.8
		Malignant N.	1,033	80.8	41.8	1,126	81.3	45.5	1,409	78.2	49.0	1,476	77.2	51.9	1,540	77.5	54.7	1,649	76.7	58.5
Age	40–49	Subtotal	608	47.6	79.6	678	49.0	87.4	802	44.5	94.0	852	44.6	103.5	914	46.0	110.7	975	45.3	112.5
		CIS	111	18.3	14.5	134	19.8	17.3	170	21.2	19.9	193	22.7	23.4	213	23.3	25.8	229	23.5	26.4
		Malignant N.	497	81.7	65.1	544	80.2	70.1	632	78.8	74.1	659	77.3	80.0	701	76.7	84.9	746	76.5	86.1
		Subtotal	456	35.7	58.8	469	33.9	59.9	691	38.4	71.5	665	34.8	71.5	690	34.7	75.1	787	36.6	81.4
	50–59	CIS	89	19.5	11.5	87	18.6	11.1	154	22.3	15.9	163	24.5	17.5	142	20.6	15.4	182	23.1	18.8
		Malignant N.	367	80.5	47.3	382	81.4	48.8	537	77.7	55.6	502	75.5	54.0	548	79.4	59.6	605	76.9	62.6
		Subtotal	161	12.6	28.0	181	13.1	32.6	225	12.5	35.8	291	15.2	45.4	285	14.4	45.8	296	13.8	42.0
	60–69	CIS	36	22.4	6.3	32	17.7	5.8	55	24.4	8.8	61	21.0	9.5	77	27.0	12.4	65	22.0	9.2
		Malignant N.	125	77.6	21.7	149	82.3	26.8	170	75.6	27.1	230	79.0	35.9	208	73.0	33.5	231	78.0	32.8
		Subtotal	53	4.1	14.9	57	4.1	15.9	83	4.6	19.4	104	5.4	23.1	97	4.9	21.5	92	4.3	18.5
	70	CIS	9	17.0	2.5	6	10.5	1.7	13	15.7	3.0	19	18.3	4.2	14	14.4	3.1	25	27.2	5.0
		Malignant N.	44	83.0	12.4	51	89.5	14.2	70	84.3	16.4	85	81.7	18.9	83	85.6	18.4	67	72.8	13.5
		Subtotal	765	59.9	35.4	813	58.7	38.3	1,050	58.3	42.6	1,136	59.4	46.8	1,195	60.2	50.2	1,331	61.9	56.0
Results of screening	Normal	CIS	129	16.9	6.0	152	18.7	7.2	208	19.8	8.4	246	21.7	10.1	253	21.2	10.6	314	23.6	13.2
		Malignant N.	636	83.1	29.4	661	81.3	31.2	842	80.2	34.2	890	78.3	36.7	942	78.8	39.6	1,017	76.4	42.8
	Benign lesion	Subtotal	513	40.1	167.5	572	41.3	162.4	751	41.7	183.7	776	40.6	185.9	791	39.8	180.4	819	38.1	186.8
		CIS	116	22.6	37.9	107	18.7	30.4	184	24.5	45.0	190	24.5	45.5	193	24.4	44.0	187	22.8	42.6
		Malignant N.	397	77.4	129.6	465	81.3	132.0	567	75.5	138.7	586	75.5	140.4	598	75.6	136.4	632	77.2	144.1

*IR* incidence rate of breast interval cancer, the detected breast cancer patient among 100,000 negatives of breast cancer screening, *CIS* Carcinoma in situ of breast, *Malignant N* Malignant neoplasm of breast
  Based on the results of breast cancer screening, the incidence rate of interval breast cancer from the normal increased from 35.4 in 2009 to 56.0 in 2014. During the same period, the incidence rate of interval breast cancer from the benign lesions increased from 167.5 to 186.8.
5. Sensitivity and specificity of screening for breast cancer
  From 2009 to 2014, the sensitivities of screening for breast cancer were 74.6, 74.1, 72.3, 73.6, 74.1, and 75.1%, respectively, and the specificities were 83.1, 85.2, 85.7, 85.4, 85.7, and 85.7%, respectively (Table 4).

**Table 4:** Sensitivity and specificity of screening for breast cancer with mammography from 2009 to 2014

			Breast cancer based on health insurance claim data				Total
			Detected		Not detected		
Screening for breast cancer with mammography	2009	Positive	3,759	74.6	503,365		507,124
		Negative	1,278		2,468,639	83.1	2,469,917
		Total	5,037		2,972,004		2,977,041
	2010	Positive	3,953	74.1	430,430		434,383
		Negative	1,385		2,472,196	85.2	2,473,581
		Total	5,338		2,902,626		2,907,964
	2011	Positive	4,696	72.3	480,664		485,360
		Negative	1,801		2,872,365	85.7	2,874,166
		Total	6,497		3,353,029		3,359,526
	2012	Positive	5,324	73.6	485,532		490,856
		Negative	1,912		2,841,889	85.4	2,843,801
		Total	7,236		3,327,421		3,334,657
	2013	Positive	5,676	74.1	468,980		474,656
		Negative	1,986		2,815,339	85.7	2,817,325
		Total	7,662		3,284,319		3,291,981
	2014	Positive	6,493	75.1	468,163		474,656
		Negative	2,150		2,815,175	85.7	2,817,325
		Total	8,643		3,283,338		3,564,681

## Discussion

This study was performed to evaluate the contributions and limitations of breast cancer screening with mammography based on the detection rate and PPV of screening for breast cancer and the incidence rate of interval breast cancer.

The crude detection rate of breast cancer screening, i.e., the crude detection rate of carcinoma in situ and malignant neoplasm, is increasing every year. On the other hand, the crude detection rate of malignant neoplasm of the breast in this study was about 7.0 –15.0% higher than that per 100,000 women over 40 in the data of the Central Cancer Registry (91.8 in 2009, 94.9 in 2010, 104.3 in 2011, and 106.3 in 2012) [1]. The detection rates of screening for breast cancer (including invasive breast cancer and ductal carcinoma in situ) employing mammography in 1996–2009 for the Breast Cancer Surveillance Consortium (BCSC) in the USA and Copenhagen and Funen in Denmark were 4.3, 6.8, and 5.8 per 1000 female screening participants, respectively.

Although there were some differences in survey time, these values were 4.4–6.7 times higher than those that detected in Korea in 2009 [21]. However, the detection rate of screening for breast cancer is influenced by the incidence of breast cancer, the age of screening participants, and the sensitivity and specificity of screening tools for breast cancer, so assessing validity based on simple comparisons has some limitations. For example, the age-standardized incidence rates of breast cancer per 100,000 breast cancer screening participants in the USA and Denmark in 2012 among women 50– 69 years old were 92.9 and 105.0, respectively, which were 2.1 and 2.3 times higher, respectively, than the incidence rate (44.7) in Korea. The ages of breast cancer screening participants were 50–69 in Denmark and 51–74 in the USA. This limited participants to the age group with relatively high incidence rates of breast cancer, but screening for breast cancer conducted by NHIS targeted all women over 40 [21]. The differences in sensitivity and specificity of screening for breast cancer are discussed below.

The PPV of suspected breast cancer increased from 20.0% in 2009 to 37.9% in 2014, and that of cases classified as deferred increased from 0.5 to 1.0% during the same period. According to the guidelines on reporting results and recommendations of the Korea National Cancer Screening Program, deferred cases are those in which a judgment of normal, benign lesion, or suspected cancer cannot be made based only on the present examination, and additional examinations, such as ultrasound or magnified views, comparison with previous mammograms, or re-examination after some period is recommended for accurate diagnosis. To overcome the limitation of the mammography and increase PPV of breast cancer screening, some technique using infrared thermography was introduced. For example, Gerasimova-Chechkina et al. [22] showed combining sparse and sometimes painful and quite uncomfortable mammography examinations with more frequent inexpensive, quick and painless infrared thermography examinations could become a very efficient routine breast cancer. For suspected breast cancer, biopsy within a short period is recommended for definite diagnosis [23]. Among the deferred cases, the rates of those treated for breast cancer within 6 months after screening were 0.5 in 2009 and 1% in 2014. For accurate diagnosis, deferred cases should be recommended to undergo additional measures, such as re-examination after 2 months or immediate ultrasound examination of the breast. However, there have been no previous reports regarding the rates at which screening recipients underwent which types of additional measures after receiving a deferred judgment.

This study showed that the incidence rate of interval cancer per 10,000 negative (normal, benign lesions) results on screening for breast cancer increased from 5.2 persons in 2009 to 7.8 persons in 2014; especially, the incidence rate of malignant neoplasm of the breast during the same period increased from 4.2 persons to 5.7 persons. With the interval cancer incidence period set to 1 year after screening, the incidence rate of interval cancer of breast cancer increased from 8.5 to 10.1, and that of malignant neoplasm of the breast increased from 6.9 to 7.8 (not shown in the Results section). There are five possible reasons why interval cancer may occur [24]. First, no sign of disease may be detected on previous screening mammogram; the lesion is new (true negative interval cancer). Second, a lesion that proves to be malignant showed benign morphological characteristics on the previous mammogram (benign interval cancer). Third, a now-known lesion is seen on the previous screen mammogram; this is an interpretive error on the part of the reader (retrospectively visible interval cancer). Fourth, a second reader may discover the lesion (single reader interval cancer); second reads in screening programs yield up to a 10% increase in cancer detection.

Breast cancer is diagnosed based on the architectural distortion, asymmetric density and irregular, speculated margins with clustered calcification on the mammogram by one radiologist who has the certificate for reading the mammogram in Korea [23]. In order to overcome the limitation of the reading by one radiologist, it is proposed to use the use computer-aided diagnostic (CAD) methods. Some studies introduced its strength [25] and showed its use during screening mammography increased the incidence of ductal carcinoma in situ, the diagnosis of invasive breast cancer at earlier stages, and increased diagnostic testing among women without breast cancer [26].

Fifth, a technically poor image may prevent the reader from discovering the abnormality; in theory, suboptimal images should not be submitted for interpretation, and those that are submitted should not be read (technical failure interval cancer). Among the 240 diagnosed interval breast cancers in Korea, 78 (32.5%) were classified as true interval breast cancer in which previous screening showed no signs of breast cancer, and the cancer had newly occurred; 78 (32.5%) as minimal signs; and 84 (35%) as missed interval breast cancer, where there was a suspicion of breast cancer, but the cancer could not be detected [27]. As the first two of the five types of interval cancer are limitations of the screening procedure itself and the remaining three types of interval cancer of the breast could have been detected at the time of screening, active quality control of the equipment and facility used for mammography, mammograms, and reading is required.

NHIS conducts quality control by introducing guidelines and performing periodic checkups on the human resources, equipment, facilities, and environments [28], as well as providing diverse types of education aimed at the radiologists and radiology technicians who read the results [29]. Previously, the incidence rates of interval cancer of the breast reported by BCSC in the USA and in Copenhagen and Funen, Denmark, were 9, 8, and 8 per 10,000 screening participants, respectively [21].

Finally, the sensitivities and specificities of screening for breast cancer using mammography did not change, with values of 74.5 and 83.1% in 2009, and 75.1% and 85.7% in 2014, respectively. Previously, the sensitivity for participants undergoing breast cancer screening for the first time were 91.8% for the BCSC, 90.5% in Copenhagen, and 92.5% in Funen, and the sensitivities for participants undergoing breast cancer screening more than twice were 82.3, 88.9, and 86.9%, respectively. The specificities for participants undergoing breast cancer screening for the first time were 83.2, 96.6, and 97.9%, respectively, while those for participants who underwent breast cancer screening more than twice were 91.6, 98.8, and 99.2%, respectively [21]. In comparing the sensitivities and specificities of screening for breast cancer according to breast-cancer related symptoms (none, meaningful symptoms, and other symptoms) prior to screening in Australia, the sensitivity and specificities for the screening group without symptoms were 75.6 and 94.9%, respectively, those for the screening group with meaningful symptoms were 80.8 and 73.7%, respectively, and those in the screening group with other symptoms were 60.0 and 95.4%, respectively [30]. Generally, mammography shows lower sensitivity in younger subjects and in those with dense breast tissue. In a study comparing parenchyma and sensitivity, the sensitivity in subjects over 50 years old decreased from 98.4% in participants with fatty breast tissue to 83.7% in those with dense breast tissue, and the sensitivity of the high-risk group with dense breast tissue decreased to 68.8% [31, 32].

The principal goal of breast cancer screening is to reduce breast cancer mortality and morbidity. Many countries monitor the individual steps throughout the entire screening process in order to ensure that the objectives of a successful breast cancer screening program with some performance indicators such as participation rate, retention rate, diagnostic interval etc. [33–35]. However, there is no formal guideline for monitoring breast screening program performance in Korea. This study would be a milestone to develop the Korean version for the guideline for monitoring breast screening program performance.

This study had a number of limitations. First, as health insurance claim data were used to detect breast cancer in the Central Cancer Registry, there may have been some differences between the actual incidences of breast cancer in the Central Cancer Registry and the cases detected in this study. In fact, the data of the Central Cancer Registry included deceased subjects, and the data from the present study excluded end stage breast cancer patients who received only palliative treatment with no anticancer treatment or surgery. However, the number of detected malignant neoplasms of the breast ranged from 97.3 to 101.1% of the value given by the Central Cancer Registry data during 2009 to 2012. In addition, as most cancer patients qualified for special case calculation, the difference between the number of the detected breast cancers in this study and the number in the Central Cancer Registry was unlikely to be large enough to significantly influence our results. Second, the detection of breast cancer may be influenced by symptoms, participation in screening, and level of exposure to risk factors such as pregnancy, breast feeding, and hormone treatment, etc. However, these factors were not taken into consideration in this study. We attempted to reduce the possibility of detection due to factors other than screening for breast cancer conducted by NHIS by limiting the period of detection of breast cancer to within 6 months after screening.

## Conclusions

For breast cancer screening using mammography to play a role in detecting breast cancer, the participation rate, especially for women in their 40s and 50s, should be higher. In addition, an environment where accurate mammography and reading can be performed, and reinforcement of quality control are required. Appropriate guidelines for deferred determination (e.g., coverage of ultrasound examination by insurance in cases of deferred determination) should be prepared. To reduce the incidence rate of interval cancer of the breast, it will be necessary to educate women after their 20s to perform self examination of the breast once a month regardless of participation in screening for breast cancer.

## Abbreviations

BCSC: Breast cancer surveillance consortium
NHIS: National health insurance service
PPV: Positive predictive value
